# GABAergic circuits underpin valuative processing

**DOI:** 10.3389/fnsys.2015.00076

**Published:** 2015-05-12

**Authors:** Dave J. Hayes

**Affiliations:** Division of Brain, Imaging and Behaviour–Systems Neuroscience, Toronto Western Research Institute, Toronto Western Hospital, University Health NetworkToronto, ON, Canada

**Keywords:** affect, appetitive, aversive, emotion, limbic, punishment, reward, valence

Affect is the fundamental neuropsychological state combining value- and arousal-related processes underpinning emotion and mood. A major goal of the emerging field of affective science is to explain the mechanisms of valuation within the brain. A core network of brain activity is seen across mammals in response to appetitive or aversive stimuli, and appears to be largely independent of stimulus modality (Bissonette et al., 2014; Hayes et al., 2014a). However, the underlying mechanisms of valuation (i.e., appetitive- and aversive-related brain activity) are unclear, and there is particularly little information about how these two valuative networks interact. One candidate which is likely central to the activity of both networks is the neurotransmitter γ-aminobutyric acid (GABA). Here, I briefly discuss some of the evidence pointing to GABA as a central player in mediating appetitive and aversive activity throughout the brain. The broader implication is that the role of GABA in valuative processing may be at the heart of affective regulation, and thus also important for a wide variety of psychological phenomena, from emotion (Stan et al., 2014) and impulsivity (Hayes et al., 2014b) to sense of self (Wiebking et al., 2014a,b).

## Keys to understanding GABA circuitry

An exploration of GABA in appetitive and aversive behavior began following its identification in the mammalian brain (Roberts and Frankel, 1950). Although central dopamine was discovered seven years later, many barriers to GABA-related research—including its relative ubiquity throughout the brain, the robust effects of GABAergic drugs administered systemically (e.g., which can easily lead to seizures or immobility), and little knowledge about GABAergic neurocircuitry—has led to a much greater understanding of dopamine in this context (Iversen and Iversen, 2007). Early advances in rodents were nonetheless made delineating key roles for GABA in consummatory behavior, stress, and anxiety (Kelly et al., 1977; Biggio et al., 1990). Studies such as these revealed that beyond dopamine, GABA was involved in mediating motivated behaviors in widespread, but regionally-selective ways (Kelly et al., 1977), and that dynamic cortical and subcortical changes to GABAergic microcircuits were involved (Biggio et al., 1990).

**Figure 1:**
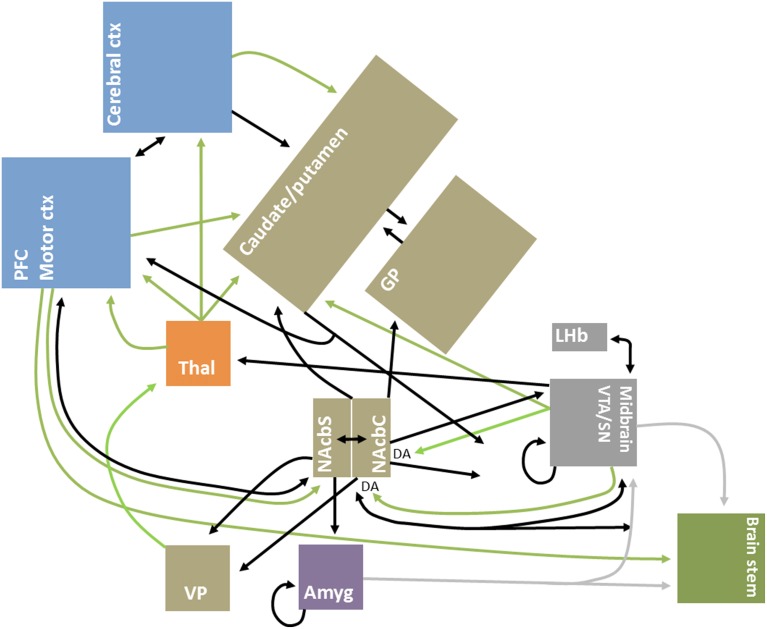
**Simplified schematic diagram of GABAergic circuitry underlying valuative processing**. Inhibitory connections, mainly GABAergic, are indicated by black arrows; excitatory connections, which are glutamatergic unless otherwise indicated, are indicated by green arrows. Brain regions are grouped by neocortex (blue), basal ganglia (brown), thalamus (orange), midbrain/mesencephalon (gray), amygdala (purple) and brainstem (green) for illustrative purposes. Adapted from Dalley et al. (2011), Squire et al. (2012), and Nieh et al. (2013).

Improved mapping of the extensive brain GABAergic circuits (illustrated in Figure 1), coupled with technological advances in detection and manipulation, have partly driven the recent focus of the role of GABA, and its sister excitatory neurotransmitter glutamate, in value-related processing. Moreover, structural advances have continued steadily, from the identification of key GABAergic hubs, such as the basal ganglia and nucleus accumbens (Groenewegen and Russchen, 1984), to more recent elaborations on the nature of inter- and intra-regional short and long-range GABAergic connections (Caputi et al., 2013). GABA circuits are uniquely situated as both local communicators and whole-brain integrators, given their dynamic control over excitatory and inhibitory signal conduction through axo-dendritic and astrocytic synapses (Frola et al., 2013) and their role in broader oscillatory and synchronistic activities (Melzer et al., 2012).

GABAergic interneurons, particularly parvalbumin-containing, are fundamental drivers of cortical oscillations, which emerging research suggests may be a fundamental context-dependent mechanism for intra- and inter-regional communication (Sohal, 2012; Jadi and Sejnowski, 2015). For instance, beyond the hippocampus and select regions of the neocortex, where these oscillations are better studied, there is also evidence for GABA-driven oscillatory synchronization within the striatum (Sharott et al., 2009)—a hub region connecting the cortex to the basal ganglia and heavily involved in motivation and valuative processing. Moreover, there is evidence that GABA cells are necessary for sustained reward-related signaling, as noted in a study of reversal learning in mice with decreased levels of prefrontal cortical GABAergic interneurons (Bissonette et al., 2015). Going forward, I briefly discuss a sample of recent studies which support the fundamental role of GABA in valuative processing and highlight future directions which will likely contribute to advances in this area.

## GABAergic microcircuits regulate valuative networks

The present focus on GABA is not intended to ignore the important role played by other neurotransmitters. GABA-glutamatergic dynamics are fundamentally important for a complete understanding of circuit dynamics, as has been underscored by findings of neuronal co-release in value-related regions (Root et al., 2014) and multimodal neuroimaging studies in humans (Duncan et al., 2014). Nonetheless, GABA likely plays a unique role in neural control as, for instance, local and long-range GABAergic projections are highly diffuse and GABAergic synapses can precisely target the dendritic shafts of pyramidal cells, allowing for earlier neuronal signal gating in comparison to glutamatergic synapses (Chiu et al., 2013). Moreover, some GABAergic projections are noted to bypass the typical brainstem-thalamo-cortical and cortico-basal ganglia-thalamo-cortical connections, including for instance the direct meso-cortico, meso-limbic and pallidal-cortical pathways (Cohen et al., 2012; Nieh et al., 2013; Saunders et al., 2015)—see Figure 1 for illustrative purposes.

Studies investigating neurotransmitter involvement in combined appetitive and aversive processing are limited in general and sparser for GABA (Hayes et al., 2014a). Existing studies in rodents using combined electrophysiological and optogenetic techniques have shown that the majority of ventral tegmental area (VTA) cells respond to value-related stimuli. This is interesting as one-third to half of VTA cells are GABAergic (Swanson, 1982) and may also include cells which co-release dopamine and GABA (Tritsch et al., 2012). While dopaminergic cells typically increase activity to appetitive stimuli as might be expected, some GABAergic cells can increase activity in response to aversive stimuli, be modulated by reward cues (Cohen et al., 2012), and also appear to help signal expected rewards (Lammel et al., 2012). Many of these cells are flexibly responsive to both appetitive and aversive cues, suggesting that they respond to the learned value of the stimulus and not its static properties (Kim et al., 2010). Networks of intra-VTA GABAergic cells, however, show increased correlations to theta band power in response to appetitive, but not aversive, cues (Kim et al., 2012) and so might be involved in the integration of appetitive networks needed to learn about, and maintain, reward-related behaviors such as electrical brain self-stimulation (Steffensen et al., 2001; Lassen et al., 2007).

Though the nucleus accumbens is comprised almost entirely of short- and long-range GABAergic projections and is a high-density region tasked with integrating motor, sensory, and valuative/motivational signals (Mogenson et al., 1980), it has been underexplored in this context (Carlezon and Thomas, 2009; Hayes et al., 2014a,b). Feeding studies have shown that presumably inhibiting the GABAergic cells of the accumbens shell corresponds to increases in appetitive feeding behaviors (Stratford and Kelley, 1997). Others have identified a rostrocaudal gradient in the shell, whereby GABA_A_ receptor activation in the rostral shell leads to increases in appetitive feeding, conditioned place preference and sucrose responding, and caudal activation results in aversive, defensive, behaviors (Reynolds and Berridge, 2002). Although our group found similar orexigenic effects of GABAergic drugs in the rostral shell, we noted a clear increase and decrease in electrical brain self-stimulation following intra-accumbens GABA_A_ receptor blockade and stimulation, respectively, at the same injection sites (Hayes et al., 2011). These seemingly opposing results suggest that although GABAergic accumbens microcircuits are fundamental to valuative processing, subtle differences in their activation are likely involved in differentiating responses to different kinds of appetitive and aversive stimuli. One clear possibility is that valuative GABAergic signaling is context-dependent on the temporal interplay with other biochemicals, such as glutamate (Clements and Greenshaw, 2005; Richard and Berridge, 2011).

The evidence for GABA as a central regulator in valuative processing is not simply limited to the VTA or accumbens. For instance, the GABAergic lateral habenula inhibits VTA-related medial prefrontal cortex dopaminergic projections and local GABAergic cells which are both tied to aversive processing and behavioral output (Lammel et al., 2012; Shabel et al., 2012). The activation of nucleus accumbens GABAergic cells are also known to inhibit ventral pallidal activity (Wang et al., 2014), which is an area that contributes to changes in valuative responding (Tindell et al., 2004). For example, inactivation of pallidum by stimulating inhibitory GABA_A_ receptors results in the elimination of reward-related saccade responding in rhesus monkeys (Tachibana and Okihide, 2013). Interestingly, a recent study suggested that increases in feeding following intra-pallidal, but not intra-accumbens, GABA_A_ receptor antagonism corresponded to a specific “fat craving” signal instead of general increases in appetitive activity (Covelo et al., 2014). Aversion-related activity may also involve GABAergic inhibition of infralimbic cortex in rats, a homolog to the primate medial prefrontal cortex, as has been demonstrated by showing that pain-related GABAergic inhibitions in the prefrontal cortex are reversed following the local injection of GABA_A_ receptor antagonists (Ji and Neugebauer, 2011)—though infralimbic activation in the absence of pain may also be anxiogenic (Bi et al., 2013). Moreover, GABA_A_ receptor activation in the infralimbic cortex increases impulsive responding (Murphy et al., 2012), while activation in the prelimbic cortex reduces aversive behaviors such as fear-potentiated startle and freezing (Almada et al., 2015).

Beyond the regions of the extended amygdala noted above (e.g., nucleus accumbens shell, habenula), the amygdala itself is one region that deserves a brief note. Although its role in processing aversive stimuli is well-established, our understanding of its role in appetitive encoding is relatively recent —it is also considered mainly as a singular structure in human studies, though it is known to be made up of a number of uniquely-connected nuclei (e.g., central, basolateral, and lateral) comprised of differing cell types (Phelps and LeDoux, 2005; Janak and Tye, 2015). Few studies have looked at how this region processes both appetitive and aversive stimuli and none have focused on GABAergic cells. Studies in primates and rodents using single cell basolateral and central area recordings showed that at least half of the cells sampled were value-responsive, sensory modality-independent, consisted of general responders and those with preferences for either appetitive or aversive stimuli, and that there was no clear anatomical distribution for such cells across each subregion (Paton et al., 2006; Belova et al., 2007, 2008; Shabel and Janak, 2009). At least one study has also shown the importance of safety signaling for cells throughout the basal amygdala, identifying subpopulations of cells that respond to combinations of value and safety cues (Sangha et al., 2013). Of further interest, recent reports have described long-range GABAergic hippocampal- and intra-amygdalar projections likely involved in valuative processing (Bienvenu et al., 2015; McDonald and Zaric, 2015).

Taken together, and as underscored by Figure 1, these studies support the hypothesis that interconnected GABAergic microcircuits are a binding feature of valuative processing. Indeed, these circuits may be at the heart of whole-brain reinforcement or valuative networks (English et al., 2011; Vickery et al., 2011). Moreover, they likely also contribute to the intraregional integration and differentiation of appetitive and aversive signals (Hayes et al., 2014a).

## Future considerations

A corollary of the hypothesis above is the emphasis on undiscovered intra- and inter-regional value-related GABAergic signaling throughout the brain. In this vein, our group showed in a human multimodal neuroimaging study that GABA_A_ receptors in medial prefrontal cortex are negatively correlated to aversive signals in both the medial prefrontal region itself as well as distant sensorimotor clusters, but that GABA_A_ receptors within different clusters of sensorimotor cortex respond differentially to the context of aversive stimuli (Hayes et al., 2013). Moreover, we reviewed the human and rodent literature on impulsivity and GABA, and concluded that GABAergic networks throughout at least cortico-basal ganglia regions acted as a common substrate of impulsive behaviors (Hayes et al., 2014b).

We believe that affective/valuative networks are at the core of many psychological phenomena, from emotion (Stan et al., 2014) and impulsivity to sense of self (Wiebking et al., 2014a,b), and may even be fundamental to the initial recruitment of cognitive control (Inzlicht et al., 2015). Indeed, some neuroimaging studies have identified a common wide-spread network of regions which may be common to these processes (Northoff and Hayes, 2011; Amft et al., 2015). Future studies using complex multimodal neuroimaging approaches will be necessary to elaborate and connect the present neural and biochemical findings in humans (Duncan et al., 2014). These should also include *in vivo* neuroanatomical explorations of affective circuitry white matter, by for instance using recent advancements in multitensor tractography (Chen et al., 2014).

Conceptually, it is important to note that when discussing a “system” of any sort in neuroscience (e.g., GABAergic, valuative) one is often taking a broad sum-of-parts operational definition. Because the entirety, and mechanistic underpinnings, of such systems are incomplete, and cannot be fully understood in isolation, these terms become placeholders for our dynamic knowledge (see LeDoux, 2012; Gross and Barrett, 2013; Hayes et al., 2014a for related discussions). For example, future research will have to continue to identify clusters of GABAergic cells which make up value-processing microcircuits as well as their connections to other value- and non-value related clusters, including other cell types, such that a better understanding of their true function becomes clearer (and probably resulting in clearer delineations between multiple “systems”). Analogous advances in network neuroscience have been made to identify many major nodes/hubs (i.e., clusters), edges (i.e., connections), and the interactions within and between such brain networks (Behrens and Sporns, 2012)—while most of this work is being done in humans, progress on the vast animal literature has also been made (Ikemoto, 2010). At this point, the greatest advances at the molecular-cellular level of understanding are likely being made through the identification and spatiotemporal electrochemical characterization of value-related microcircuits, for instance in the traditional mesocorticolimbic circuit (e.g., Nieh et al., 2013; Lammel et al., 2014). Indeed, the bulk of information connecting behavior to underlying mechanisms is confined to this value-related circuit, with disparate results for other areas. Moreover, behavioral-cellular/neurochemical connections related to GABA have been mainly restricted to single regions, such as the VTA, nucleus accumbens, and areas of the prefrontal cortex, although recent experiments have focused increasingly on inter-regional interactions (Lammel et al., 2012; Shabel et al., 2012; Hayes et al., 2013; Wang et al., 2014).

Going forward, future studies will also need to clearly answer the question of how brain areas within similar affective networks parse aversion- and appetitive-related neural signals, while also providing mechanisms for fast intraregional communication. Recent reviews of the human and animal literature have provided some insight to this question (Bissonette et al., 2014; Hayes et al., 2014a; Lindquist et al., 2015), but more work is needed. For instance, the significance, if any, of structures which show asymmetrical activity is unclear, e.g., appetitive and aversive stimuli may result in more dopamine in the right and left accumbens, respectively (Besson and Louilot, 1995). Moreover, how these neural processes are translated at the behavioral or “cognitive” level is of equal importance. Can appetitive and aversive stimuli be subjectively, consciously, experienced simultaneously (Barrett et al., 2007)? Why do some people experience painful experiences as pleasurable, and is there any connection to those who prefer to self-administer aversive stimuli rather than be alone with their own thoughts (Wilson et al., 2014)?

### Conflict of interest statement

The author declares that the research was conducted in the absence of any commercial or financial relationships that could be construed as a potential conflict of interest.

## Abbreviations

Ctx: cortex
DA: dopamine
Amyg: amygdala
GABA: gamma-aminobutyric acid
GP: globus pallidus
LHb: Lateral habenula
Motor ctx: motor cortices including premotor, supplementary motor, and frontal eye fields
NAcbC: nucleus accumbens septi core
NAcbS: nucleus accumbens septi shell
PFC: prefrontal cortex
SN: substantia nigra
Thal: thalamus
VP: ventral pallidum
VTA: ventral tegmental area.
